# Research on the carrying capacity of production, living and ecological space and its coupling coordination in Duolun County, Inner Mongolia

**DOI:** 10.1371/journal.pone.0309615

**Published:** 2024-12-31

**Authors:** A ruhan, Dongchang Liu

**Affiliations:** 1 College of Desert Control Science and Engineering, Inner Mongolia Agricultural University, Hohhot, Inner Mongolia, China; 2 Key Laboratory of Desert Ecosystem Conservation and Restoration, State Forestry and Grassland Administration of China, Inner Mongolia Agricultural University, Hohhot, Inner Mongolia, China; Dongshin University, REPUBLIC OF KOREA

## Abstract

To offer a foundational science for the land spatial planning of Beijing Tianjin sandstorm source area, the remote sensing images of Duolun County in Inner Mongolia from 2000 to 2020 were used to obtain the spatial information of production, living and ecological space(PLES). In order to construct the index system of the carrying capacity of the PLES, 24 indicators were chosen from the perspectives of ecological space, living space, and production space. AHP method, TOPSIS Model with entropy combination weight, coupling coordination model and obstacle degree model are used to analyze the coupling coordination scheduling and obstacle degree of the PLES carrying capacity. The findings exhibited that (1) the distribution area of ecological space and production space decreased, while the living space area increased slightly; (2) The carrying capacity of production space showed a weak growth trend, the carrying capacity of production space, living space and the PLES space showed a weak downward trend; (3) The coupling degree and coupling coordination degree between two spaces show a slow downward trend; (4) The obstacle degree of the carrying capacity of production space and living space has increased, while the carrying capacity of ecological space is decreasing. Livestock carrying capacity is the most important obstacle restricting the carrying capacity of production space. The scale of construction land has the greatest obstacle to the carrying capacity of living space, the degree of desertification has the greatest obstacle to the carrying capacity of ecological space, and soil organic matter has the greatest obstacle to the comprehensive carrying capacity of the PLES.

## 1 Introduction

The PLES is composed of production space, living space together with ecological space [1], productive space is a space whose primary function is to provide service, agricultural and industrial products [2], living space is the space that provides carrying and ensuring human settlement function [3], and ecological space is the space that provides ecological products and ecological services [4]. Healthy the PLES enables all spaces to achieve common progress [5] and finally achieve the best spatial carrying capacity [6]. With the continuous advancement of the process of land space development [7], human demand for production space and living space is gradually increasing [8], which leads to the imbalance of the proportion of production space, living space and ecological space structure, and also leads to the problems of ecological environment deterioration [9], frequent natural disasters, and intensified energy crisis [10]. The problem of the PLES is the uncoordinated and unbalanced problem between the PLES [11], and seriously affects the regional sustainable development [12]. The carrying capacity of the PLES refers to the carrying capacity of the PLES system to human activities and external interference under the premise of regional sustainable development, reflecting the carrying strength of the PLES to human activities, and its carrying capacity evaluation is served as the cornerstone of sustainable development. Currently, PLES’s carrying capacity primarily focused on the carrying capacity of cities [13], urban agglomerations [14], rural pastoral areas [15], water areas [16], ecological space [17], production space [18], and construction land [19]. The research methods mainly include analytic hierarchy process [20], entropy weight method [21], TOPSIS model [22], comprehensive index evaluation [23], and lack of attention to the carrying capacity of the PLES in villages. Although the scope of the village is small, it has a strong role in promoting the optimization of the spatial carrying capacity of rural PLES [24]. Duolun County in Inner Mongolia is located in the typical ecological fragile area in the middle of Beijing Tianjin sandstorm source area [25]. The land space development is increasingly active, and the spatial structure also changes [26]. How to coordinate the relationship among production space, living space and ecological space has become a serious problem for its regional sustainable development. The land use classification data were extracted by using remote sensing images and DEM from 2000 to 2020, and the temporal and spatial distribution properties of PLES were analyzed. From the perspective of production, life and ecological space, 21 indicators, including population density, grain and soybean production, livestock carrying capacity, primary industry, secondary industry, tertiary industry, GDP, elevation, topographic relief, road network density, total population, construction land scale, per capita disposable, per capita GDP, precipitation, soil texture, soil thickness, soil organic matter, soil erosion, desertification degree, vegetation coverage, were chosen to develop the assessment index system for the PLES carrying capacities and to investigate the spatiotemporal evolution of the PLES carrying capacities in Duolun County of Inner Mongolia in the past 20 years and the obstacles to its development, It provides a theoretical reference for orderly guiding the optimization of land space and the coordinated and sustainable development of regional economy, society and environment in Beijing Tianjin Hebei region and even the whole country.

## 2 Materials and methods

### 2.1 Study area

Located at the south source of Hunshandake Sandy Land [27], Duolun County is the primary location for the Beijing Tianjin Sandstorm Source Project and is part of a typical agricultural pastoral ecotone [28], and is the key area for the construction of Beijing Tianjin sandstorm source project. It borders Zhenglan Banner in the west, Keshiketeng Banner in Chifeng City in the north, Guyuan County, Fengning County and Weichang County in the south, with a total area of 3863.69km^2^. The terrain is a semi-circular basin that is high around, low in the middle and the north, high in the South, and turns from southwest to northeast and then to Southeast, with an altitude of 1043-1795m. The geomorphic types are mainly low mountains, hills, valleys and depressions, piedmont sloping plains and accumulation type dunes, and 21.97% of the total area is made up of low-lying mountains. The hills belong to the Yinshan Mountain and the greater Khingan mountains. The climate of Duolun county belongs to the typical continental climate of the transition from semi-arid to semi humid in the temperate zone, mainly including chestnut soil, meadow soil, aeolian sand soil and other soil types, and typical grassland vegetation, meadow grassland vegetation, sandy vegetation, swamp vegetation and other vegetation types. The county is in charge of 65 administrative villages, 2 townships, and 3 towns.

### 2.2 Data sources

The socio-economic Figs utilized for the study were obtained from the compilation of statistical data on national economic and social development in Duolun County, Inner Mongolia, Xilingol statistical yearbook and Inner Mongolia statistical yearbook from 2001 to 2021. Geospatial data cloud (http://www.gscloud.cn/) is applied to create the 30 meter spatial resolution Aster GDEM digital elevation model. The elevation and terrain relief are obtained through the spatial analysis function of arcgis10.8 software. Remote sensing image data comes from geospatial data cloud(http://www.gscloud.cn/) Landsat 4–5 (TM) (2000–2010) and Landsat 8 (OLI) (2015–2020), respectively. The secondary classification and vegetation coverage data were extracted by envi5.3 and arcgis10.8 software. The meteorological data is from the official website of the national meteorological data center (https://data.cma.cn/) The precipitation of Duolun County and its surrounding 13 meteorological stations were collected. Institute level data center of Nanjing Institute of soil research, Chinese Academy of Sciences (https://soildata.issas.ac.cn/). The arcgis10.8 software was utilized to acquire the secondary classification data on land use, and extract the road network density, construction land scale, desertification degree and other data.

### 2.3 Research methods

#### 2.3.1 Standardization of indicators

According to the different attributes of the assessment index of the PLES carrying capacity, it is divided into positive index and negative index. The original data is dimensionless processed utilizing the range standardization approach [29], and the formula for calculation is as below:

Processing formula of positive indicator:

$${\text{D}}_{\text{ij}}=\frac{{\text{X}}_{\text{ij}}-\text{min}({\text{X}}_{\text{ij}})}{\text{max(}{\text{X}}_{\text{ij}}\text{)-min(}{\text{X}}_{\text{ij}})}$$ (1)

Processing formula of negative indicators:

$${\text{D}}_{\text{ij}}=\frac{\text{max}({\text{X}}_{\text{ij}}\text{)-}{\text{X}}_{\text{ij}}}{\text{max}({\text{X}}_{\text{ij}})-\text{min(}{\text{X}}_{\text{ij}})}$$ (2)

Where D_ij_ represents the indicator for evaluating the PLES carrying capacity; X_i_ denotes the actual value of the indicator in the j year; min(X_ij_) is its minimum value; max(X_ij_) is its maximum value; D_ij_ is the normalized value of X_ij_, D_ij_ ∈ [0,1].

#### 2.3.2 Determining the index weight of criterion layer and index layer by analytic hierarchy process

Analytic hierarchy process (AHP) is applied to identify the subjective weight of the impact factors of the criteria layer and the index layer [30]. Through the analytic hierarchy process, a judgment matrix is established by mutual judgment and comparison, and the maximum eigenvalue and eigenvector are calculated. Pass the consistency test. If it is less than 0.1, it passes the test; Otherwise, the judgment moment is reconstructed and finally the weight of each index component is determined. The higher the value, the greater the impact. The calculation formula is:

CR=CiRi (3)

$${\text{C}}_{\text{i}}=\left(\lambda_{\text{max}}-\text{n}\right)/\left(\text{n}-1\right)$$ (4)

Where C_i_ represents the value of the consistency indicator; λ_max_ denotes the largest eigenvalue of the judgment matrix; n stands for the number of indicators; R_i_ is the average random consistency index, which is obtained by querying the consistency index table. When C_R_ < 0.1, the judgment matrix satisfies the test of consistency, else the judgment matrix should be adjusted appropriately and re-analyzed.

#### 2.3.3 Determination of index weight of index layer by entropy weight technique

Entropy weight technique was exploited to identify the indicator layer’s objective weight [31], and determine the entropy value and indicator weight formula as follows:

$${\text{A}}_{\text{ij}}={\text{P}}_{\text{ij}}∕\sum_{\text{i}=1}^{\text{n}}{\text{P}}_{\text{ij}}$$ (5)

$${\text{e}}_{\text{j}}=-\frac{\text{1}}{\text{ln}\;\text{m}}*\sum_{\text{i}=1}^{\text{m}}{\text{A}}_{\text{ij}}\text{ln}\;{\text{A}}_{\text{ij}},\text{Ej}\in \left(0,1\right)$$ (6)

$${\text{W}}_{\text{j}}=\frac{\text{1-}{\text{e}}_{\text{j}}}{\sum_{\text{i}=1}^{\text{m}}\left(\text{1-}{\text{e}}_{\text{j}}\right)}$$ (7)

Where W_j_ is the weight of indicator; A_ij_ represents the percentage of the i indicator under the j indicator; e_j_ is the entropy value of the j indicator, nm is m indicators in n years.

#### 2.3.4 AHP and Entropy weight technique for determining the weight of comprehensive indicators

The AHP method focuses on subjective evaluation, while the entropy weight method focuses on objective information [32]. By considering the subjective and objective weight coefficients comprehensively, it can reduce the bias of using a single weighting method and make the results more comprehensive and reasonable. Finally, based on the subjective weight of the Analytic Hierarchy Process, weights are combined to establish the subsystem indicator layer’s comprehensive weight. The formula for calculation is:

$${\text{a}}_{\text{i}}=\frac{{\text{u}}_{\text{j}}{\text{w}}_{\text{i}}}{\sum_{\text{j}=1}^{\text{m}}{\text{(u}}_{\text{i}}{\text{w}}_{\text{i}})}$$ (8)

Where *a*_i_ is the j indicator comprehensive weight of the indicator layer; *u*_*j*_ denotes the indicator weight identified by the AHP for j indicator in the indicator layer, *w*_*j*_ stands for j indicator weight in the indicator layer identifying via the entropy weight method, and m is the number of indicators.

The target layer’s indicator weights are calculated utilizing the AHP-Entropy weight technique, whose formula is:

$${\text{S}}_{\text{i}}={\text{a}}_{\text{i}}\times {\text{d}}_{\text{j}}$$ (9)

Where S_i_ is the comprehensive weight of the i index of the target layer; d_j_ represents the j index weight of the criterion layer.

#### 2.3.5 Calculation of comprehensive evaluation index by TOPSIS model

TOPSIS model [33] is a technique for choosing the best scheme, which uses the distance between the best and worst solutions to represent the current level [34]. In the study, the carrying capacity of PLES is comprehensively evaluated in accordance with the value and weight of each evaluation index after standardization. In addition, the carrying capacity assessment value of the PLES composite space is determined by the TOPSIS model based on the weights of the index and criterion layers.

1. Construct a weighted matrix Z for the standardization of m indicators in n regions:
$$\text{Z}={\left({\text{z}}_{\text{ij}}\right)}_{\text{mn}}\;(\text{i}=1,2\ldots \text{n};\;\text{j}=1,2\ldots ,\text{m})$$ (10)
2. Determining positive and negative ideal solutions:
  Positive ideal solution ${\text{D}}_{\text{i}}^{\text{+}}$ together with negative ideal solution ${\text{D}}_{\text{i}}^{-}$ for each scheme can be identified through weighted decision matrix. In weighted decision matrix. ${\text{D}}_{\text{i}}^{\text{+}}$ and, ${\text{D}}_{\text{i}}^{-}$ represent the optimal and worst value of each index, separately, and its formula for calculation is as below:
$${\text{D}}_{\text{i}}^{\text{+}}=\sqrt{\sum_{\text{j}=1}^{\text{n}}{\left({\text{z}}_{\text{j}}^{\text{+}}-{\text{z}}_{\text{ij}}\right)}^{\text{2}}}$$ (11)
$${\text{D}}_{\text{i}}^{-}=\sqrt{\sum_{\text{j}=1}^{\text{n}}{\left({\text{z}}_{\text{j}}^{-}-{\text{z}}_{\text{ij}}\right)}^{\text{2}}}$$ (12)
3. Calculate the sample’s approximation to the ideal solution C_i_
$${\text{C}}_{\text{i}}=\frac{{\text{D}}_{\text{i}}^{-}}{{\text{D}}_{\text{i}}^{\text{+}}-{\text{D}}_{\text{i}}^{-}}$$ (13)
  Where the value range of *C*_i_ is [0,1]. The scheme is nearer the positive ideal solution the higher the value; the scheme is further from the positive ideal solution the lower the value.

#### 2.3.6 Coupling coordination model

The amount of coordinated growth within the system cannot be reflected by the coupling degree, but it may explain the correlation and intensity of the interaction of subsystems within the carrying capacity of the PLES [35]. Coupling coordination degree denotes the degree of interaction between two or more systems [36], which can reflect the interaction strength between the carrying capacity of each subsystem of the PLES, and also present how well-coordinated the PLES’s carrying capacity has developed. On the basis of the findings of the analysis of the carrying capacity of the three subsystems of the PLES in Duolun County, Inner Mongolia, the coupling coordination degree model is constructed for realizing the coupling and coordinated development of the carrying capacity of PLES. The formula is [37]:

$$\text{C}=\sqrt[{\text{3}}]{\frac{{\text{P}}_{\text{i}}*{\text{L}}_{\text{i}}*{\text{E}}_{\text{i}}}{{\left(\frac{{\text{P}}_{\text{i}}*{\text{L}}_{\text{i}}*{\text{E}}_{\text{i}}}{\text{3}}\right)}^{\text{3}}}}$$ (14)

T=αf(x)+βg(x)+λh(x) (15)

$$\text{D}=\sqrt{\text{C}*\text{T}}$$ (16)

Where C is the coupling degree, C ∈ [0,1], P_i_, L_i_, E_i_ represent the carrying capacity of PLES separately. The nearer the value of C is to 1, indicating that the higher the coupling degree between the subsystems; T is the comprehensive evaluation index of the PLES of administrative village in Duolun County; α, β, λ is each subsystem’s weight. In this study, PLES carrying capacity are equally important. Therefore, set α = β = λ = 1/3. D is the PLES coupling coordination scheduling. The greater the D value, the higher the degree of coupling coordination.

#### 2.3.7 Obstacle degree model to determine influencing factors

The obstacle factor is diagnosed through the obstacle degree model [38] to determine the major effect indicators influencing the the PLES carrying capacity in Duolun County, Inner Mongolia. The calculation formula is:

1. Calculate the factor contribution F_ij_ of each index:
$${\text{F}}_{\text{ij}}=\omega_{\text{i}}{\text{z}}_{\text{j}}$$ (17)
  Where: ω_j_ is the comprehensive weight of index j; z_j_ is the index j weight belonging to the criterion layer.
2. The obstacle degree calculation formula is:
$${\text{P}}_{\text{ij}}=\frac{{\text{F}}_{\text{j}}{\text{I}}_{\text{ij}}}{\sum_{\text{j}=1}^{\text{n}}{\text{F}}_{\text{j}}{\text{I}}_{\text{ij}}}\times \text{100\%}({\text{I}}_{\text{ij}}=1-{\text{y}}_{\text{ij}})$$ (18)
  Where: P_ij_ is the obstacle degree of the j index of the i sample, and I_j_ is the deviation degree of the index; y_ij_ is the standardized index value.

### 2.4 Construction of evaluation index system

The evaluation factors include natural factors and socio—economic factors. Considering the conditions of climate, geology and geomorphology, soil, vegetation, hydrology and economy, referring to the national regulations such as the work plan for the evaluation, monitoring and early warning of the environmental carrying capacity of land and resources, the technical criteria for the assessment of the environmental carrying capacity of land and resources [39], and combining with the actual situation of the study area, the spatial carrying capacity of Duolun county is established from the index, criterion and target layers (Table 1). The village area serves as the assessment unit to gauge the environmental carrying capacity of the PLES. The indicators are completely weighted via the analytic hierarchy process and Delphi method.

**Table 1:** Index system and index weight of the PLES carrying capacity in Duolun County.

Target layer	Criterion layer	weight	Indicator layer	Indicator variable	Indicator attribute	Index weight				
						2000	2005	2010	2015	2020
carrying capacity of the PLES	Production space	0.182	Population density	X_1_	+	0.0008	0.0202	0.0363	0.016	0.0732
			Grain beans produce grain	X_2_	+	0.0014	0.0143	0.0159	0.0067	0.0136
			Carrying capacity	X_3_	+	0.0913	0.0211	0.083	0.0252	0.0337
			Primary industry	X_4_	+	0.0151	0.0637	0.005	0.0111	0.034
			Secondary industry	X_5_	+	0.0096	0.0055	0.0017	0.0178	0.0078
			Tertiary industry	X_6_	+	0.0471	0.0149	0.0024	0.0105	0.0047
			GDP	X_7_	+	0.0165	0.0423	0.0377	0.0945	0.0183
	Living space	0.091	Elevation	X_8_	-	0.0011	0.0012	0.0014	0.0011	0.0012
			Relief	X_9_	-	0.0012	0.001	0.0012	0.0009	0.0014
			Density of road network	X_10_	+	0.0204	0.0268	0.0279	0.0212	0.0355
			Total population	X_11_	+	0.0023	0.0174	0.0028	0.0202	0.0038
			Scale of construction land	X_12_	+	0.0032	0.0219	0.0322	0.0273	0.0261
			Per capita disposable	X_13_	+	0.0163	0.0147	0.0151	0.0091	0.0116
			Per Capita GDP	X_14_	+	0.0464	0.0078	0.0104	0.0111	0.0038
	Ecological space	0.727	Precipitation	X_15_	+	0.4389	0.2052	0.2886	0.2838	0.3627
			Soil texture	X_16_	+	0.0351	0.0736	0.063	0.051	0.0787
			Soil thickness	X_17_	+	0.0218	0.0397	0.034	0.0275	0.0427
			Soil organic matter	X_18_	+	0.1948	0.3346	0.2863	0.2318	0.1371
			Soil erosion	X_19_	-	0.0041	0.0342	0.0173	0.0129	0.0165
			Degree of desertification	X_20_	-	0.003	0.0157	0.0101	0.0065	0.0075
			Vegetation coverage	X_21_	+	0.0296	0.0242	0.0277	0.1138	0.0861

## 3 Result

**Fig 1:**
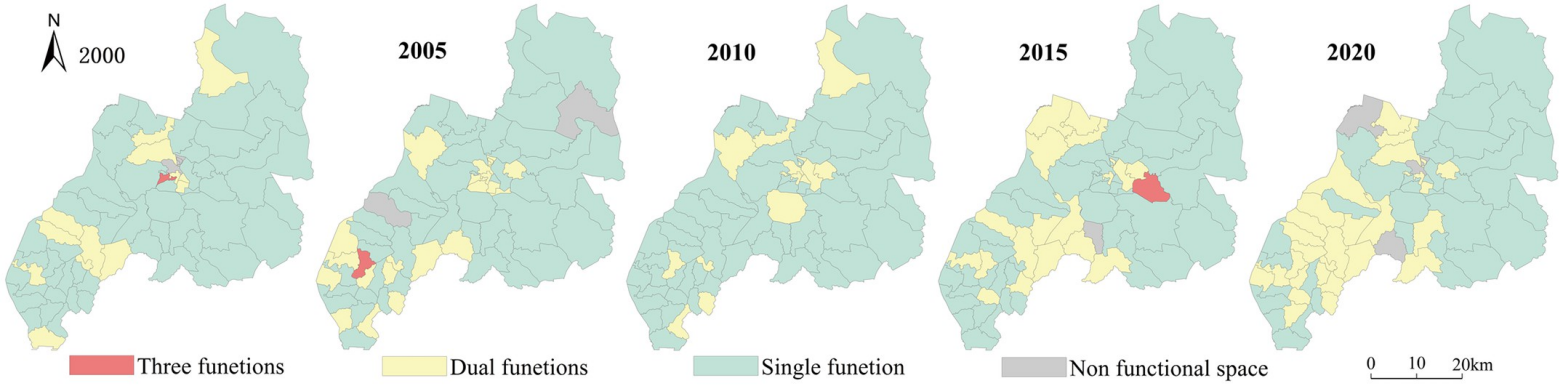
Function combination map of the PLES in Duolun County, Inner Mongolia.

**Fig 2:**
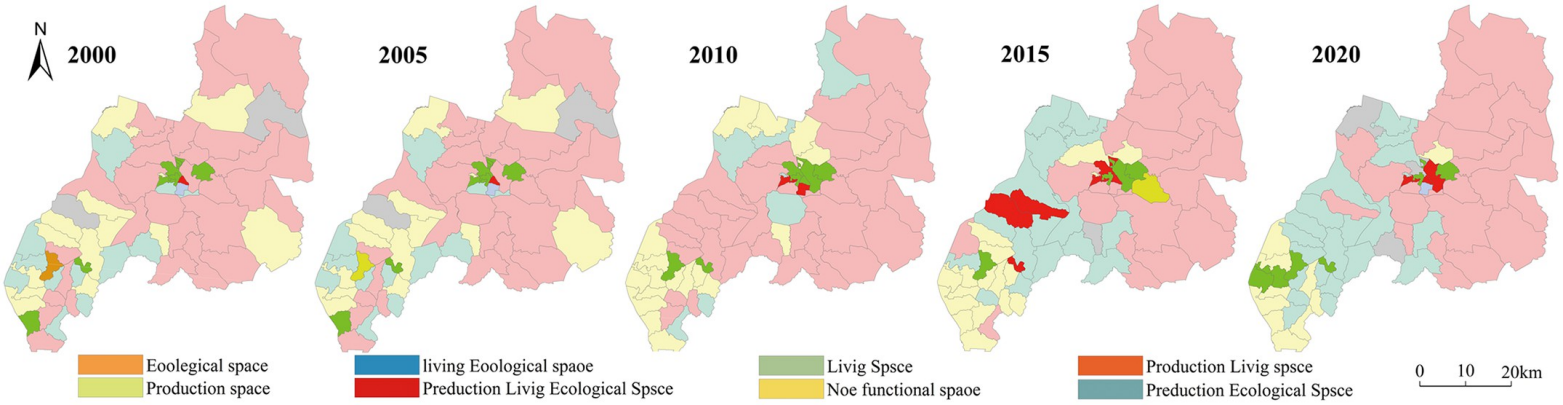
Spatial distribution map of the PLES function in Duolun County.

According to Du Lai ’s [39] classification method of the spatial function of production, living and ecological space, the strength of the spatial function of the land use types in Duolun County, Inner Mongolia, is divided into four grades: 0, 1, 3 and 5, which are translated into grid data with a spatial resolution of 500 meters. Using the method of landscape overlay analysis, the grid with the function value of PLES higher than the average value of all patches is designated as 1, and the grid with the function value lower than the average value is designated as 0. A grid value of 1 indicates a strong function of the PLES. The function values of production space, living space and ecological space are 0, indicating that the PLES is weak and can be considered as non functional areas. Through spatial superposition, the multifunctional index of the PLES function can be obtained. If the multi-function index is 1, it indicates that the region is a single function of the PLES; The multi-functional index is greater than 1, indicating that the area is a multi-functional area of the PLES. Using arcgis10.8 software and the distribution map of 65 administrative villages in Duolun County, the function combination map of the PLES (Figs 1 and 2) was obtained.

### 3.1 Spatial and temporal changes of the PLES

Fig 1 displays that the area of single function area in the the PLES function of Duolun County, Inner Mongolia, makes up 86.30% of the land’s total area. The area of ecological spatial distribution in the single function area is relatively large and concentrated in the central and eastern regions. The ecological spatial distribution area decreased from 86.3% in 2000 to 69.54% in 2020, and its spatial distribution characteristics decreased year by year from the hilly area in the southwest to the dune area in the northeast. The area dominated by dual functions is concentrated in the western part of the county seat, and its area is increasing year by year, from 505.3km^2^ in 2000 to 1031.40km^2^ in 2020, will make up 26.6% of the entire land area, in 2020. The proportion of the three functional areas is very small, and only the three functional areas in 2005 and 2015. In 2005, the three functional areas were distributed in Dabeigou village in the southwest of Duolun County. Dabeigou village is a typical area combining agriculture and animal husbandry. Planting industry is its leading industry, and it is an important main grain production area in Duolun County, and the risk of soil erosion disaster is small. In 2015, the North Village in the southeast of Duolun County had the three functional sections, where the proportion of cultivated land, grassland and forest land was small. Since 2000, Duolun County has successively implemented ecological construction projects such as the control of wind and sand sources in Beijing and Tianjin, the conversion of agricultural land to forests, the afforestation of one million mu of Pinus sylvestris var. mongolica, and the construction of a large-scale forest farm in Hunshandake sandy land, it has increased the amount of forest land in the county’s center and northern regions, reversed the enormous area of desertified land in those regions, and broadened the range of ecological spatial distribution, and reduced the area of production spatial distribution.

Fig 2 reveals that the function combination of the PLES is primarily production ecological function combination. The ecological space is concentrated in the central and northeast areas of Duolun County, and its area has decreased from 68.11% in 2000 to 60.78% in 2020. The production space is concentrated in the southwest of Duolun County, and its area has decreased from 18.19% in 2000 to 7.83% in 2020. The production − ecological function combination is concentrated in the cultivated land, grassland and forest areas in the south, and its area has increased from 11.70% in 2000 to 23.47% in 2020. The combination of production − living functions is focused on county’s southwest and center, as well as the vicinity of the urban built-up districts, with a small area. The production life ecological function combination is concentrated in the central area of the county, and its area is very small. This shows that the land spatial versatility gradually increases from the northern Desertified Area to the southwest. Through the ecological treatment project, the vegetation coverage of the whole county has been significantly increased, the output value of the primary industry on both sides of the river in the middle of the county has been significantly increased, the number of livestock carrying capacity in the middle of the county has been controlled, and the steady development of the production space functional area and the production ecological space functional area has been promoted.

### 3.2 Features of PLES carrying capacity in terms of space and time

**Fig 3:**
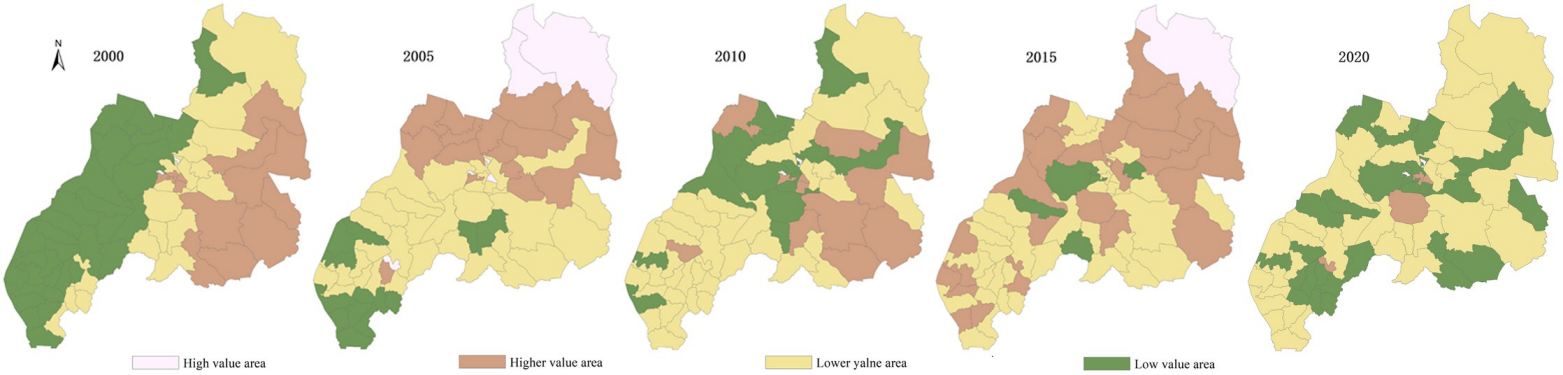
Distribution map of production space carrying capacity in Duolun County, Inner Mongolia.

**Fig 4:**
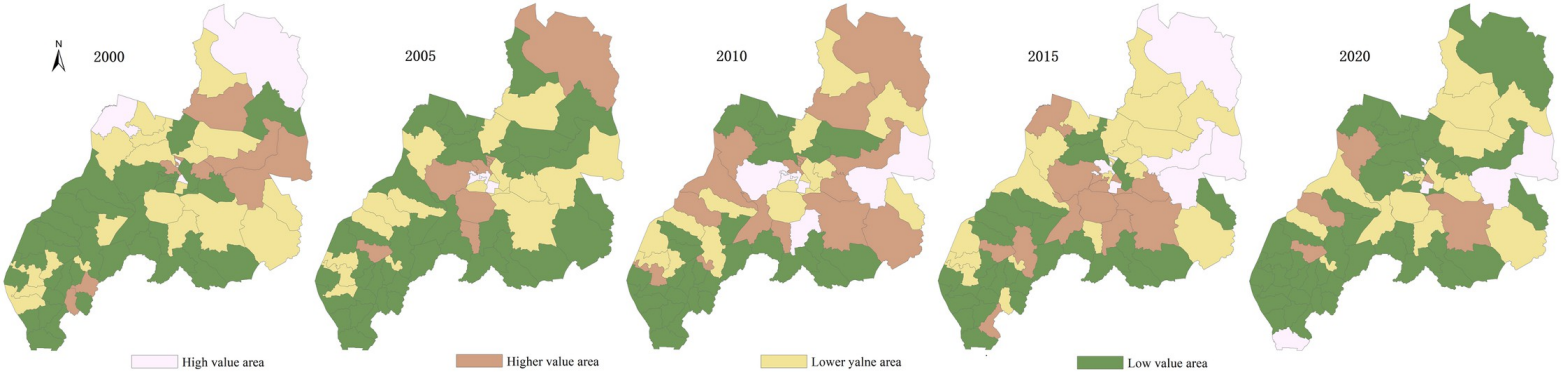
Distribution map of living space carrying capacity in Duolun County, Inner Mongolia.

**Fig 5:**
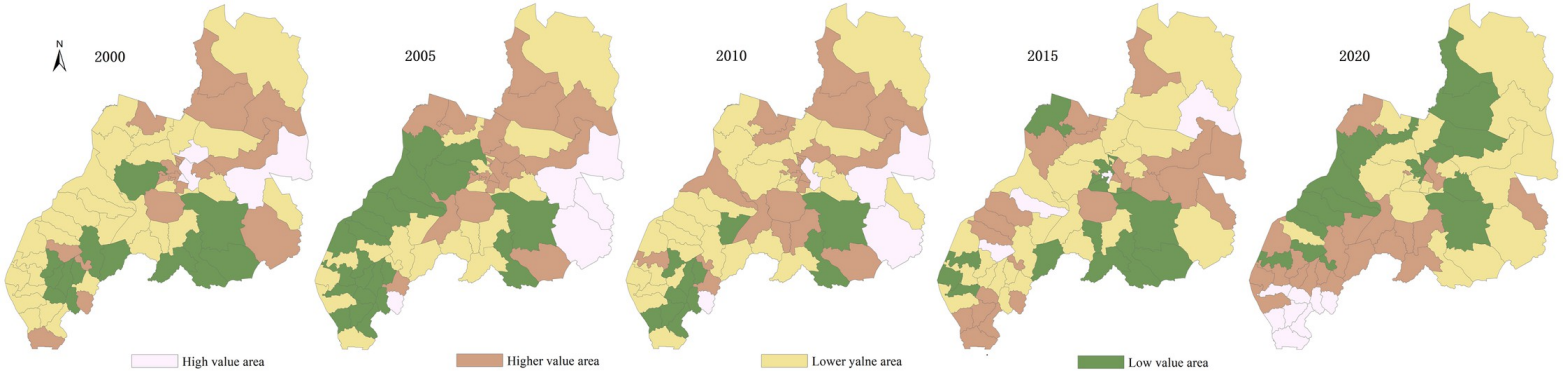
Distribution map of ecological space carrying capacity in Duolun County, Inner Mongolia.

**Fig 6:**
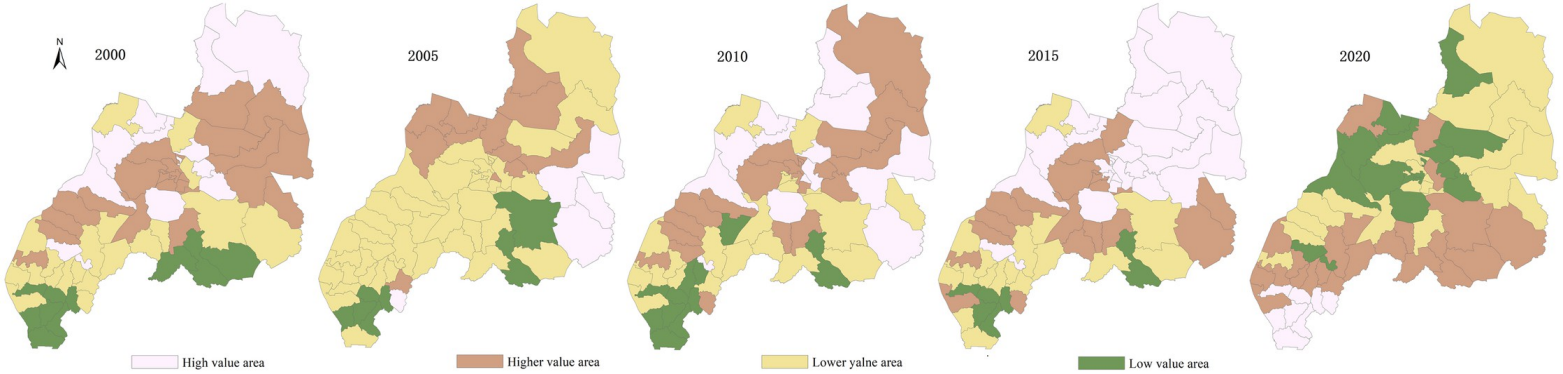
Distribution map of PLES carrying capacity in Duolun County, Inner Mongolia.

**Fig 7:**
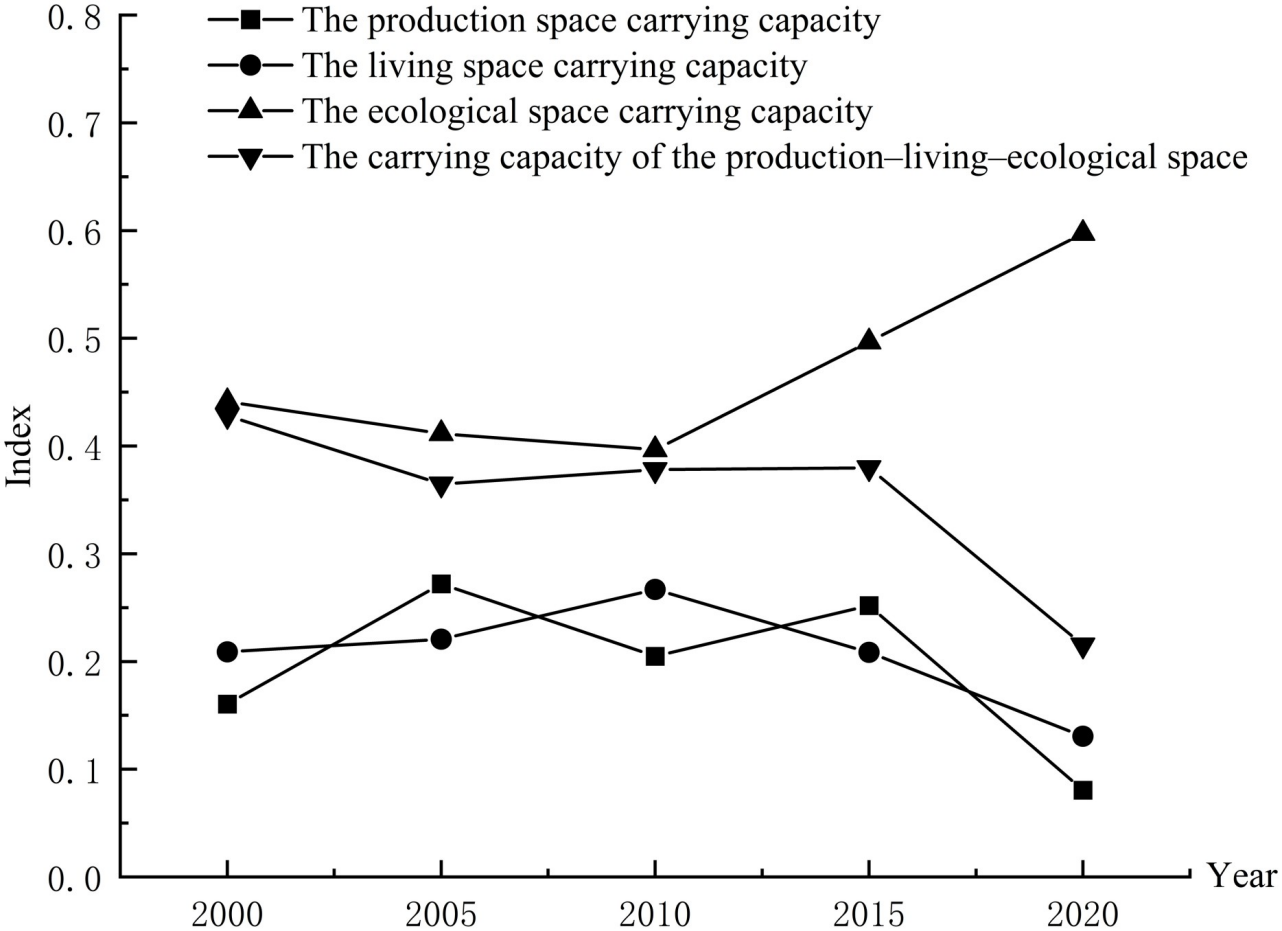
Discount chart of PLES carrying capacity.

Using ArcGIS10.8 natural breakpoint classification method, the evaluation values of the PLES carrying capacity in Duolun County, Inner Mongolia, from 2000 to 2020 are divided into five levels: high-carrying capacity, higher-carrying capacity medium—carrying capacity, lower-carrying capacity and low-carrying capacity (Figs 3–6), and the average coupling capacity of 65 administrative villages in Duolun County in Inner Mongolia is calculated (Fig 7).

#### 3.2.1 Carrying capacity characteristics of production space

It can be seen from Fig 3 that the average production space carrying capacity of Duolun County in 2000 was 0.16, and the production space carrying capacity showed the characteristics of high in the southeast and low in the northwest. The lower coupling capacity of each coupling capacity area was the largest, 1444.94km^2^, accounting for 37.4% of the total land area of Duolun County, mainly distributed in the central region. The second is the area with low coupling capacity, which is 1212.39km^2^, accounting for 31.4% of the total land area of Duolun County, mainly distributed in the western region. The distribution area of high coupling capacity is the lowest, accounting for only 0.09% of the total land area, which is distributed in the middle of the county; In 2005, the average production space carrying capacity of Duolun County was 0.27, and the production space carrying capacity was higher in the north than in the south. The lower coupling capacity of each coupling capacity area had the largest spatial distribution area, 1766.85km^2^, accounting for 45.7% of the total land area of Duolun County, which was widely distributed in the central and southern areas of the county. The second is the area with higher coupling capacity, which is 1114.38km^2^, accounting for 28.8% of the total land area of Duolun County, and is distributed in the north and middle of the county; In 2010, the average production space carrying capacity of Duolun County in Inner Mongolia was 0.20, and the spatial distribution area of the lower coupling capacity was the largest, 2036.68km^2^, which was distributed in the northwest region, accounting for 52.7% of the total area; In 2015, the average production space carrying capacity of Duolun County in Inner Mongolia was 0.25, and the area of higher coupling capacity and higher coupling capacity in each coupling capacity area was 3204.74.30km^2^, accounting for 83% of the total land area of Duolun County, which was widely distributed; In 2020, the average carrying capacity of production space in Duolun County was 0.08. The area with lower carrying capacity accounted for 2515.73km^2^, accounting for 65.1% of the total land area in Duolun County, and the area with higher carrying capacity and high carrying capacity accounted for 2.57%.

From 2000 to 2020, the average production space carrying capacity of Duolun County in Inner Mongolia decreased from 0.16 in 2000 to 0.08 in 2020.

#### 3.2.2 Carrying capacity characteristics of living space

It can be seen from Fig 4 that the average carrying capacity of living space in Duolun County in 2000 was 0.208. The carrying capacity of living space in the Northeast was higher than that in the West. The spatial distribution areas of low coupling capacity and low coupling capacity in each coupling capacity area were 1570.20km^2^ and 1224.11km^2^ respectively, accounting for 72.3% of the total land area of Duolun County; In 2005, the average carrying capacity of living space in Duolun County was 0.221. The carrying capacity of living space in the middle was higher than that in the north and south sides of the country. The area with low carrying capacity was the largest, 2117.20km^2^, accounting for 55% of the total land area of Duolun County, which was widely distributed in the middle and south of the county. The second is the space area with lower coupling capacity, which is 1043.57km^2^, accounting for 27% of the total land area of Duolun County, and is distributed in the central area of the county; In 2010, the average carrying capacity of living space in Duolun County of Inner Mongolia was 0.27, and the higher coupling capacity of living space had the largest spatial distribution area of 1503.67km^2^, accounting for 39% of the total area. The second is the space area with low coupling capacity, which is 1174.60km^2^, accounting for 31% of the total land area of Duolun County, and should be distributed in the central and southern areas of the county; In 2015, the average carrying capacity of living space in Duolun County of Inner Mongolia was 0.21, and the area with lower carrying capacity was the largest, 1223.11km^2^, accounting for 31.7% of the total land area of Duolun County, which was concentrated in the central region; In 2020, the average carrying capacity of living space in Duolun County was 0.13, and the areas of low coupling capacity and low coupling capacity were 2088.92km^2^ and 1139.71km^2^ respectively, accounting for 83.6% of the total area.

From 2000 to 2020, the living space carrying capacity of Duolun County in Inner Mongolia decreased from 0.208 in 2000 to 0.131 in 2020, and the living space carrying capacity gradually decreased from northeast to southwest of the county.

#### 3.2.3 Carrying capacity characteristics of ecological space

It can be seen from Fig 5 that the average ecological spatial carrying capacity of Duolun County in 2000 was 0.441, and the spatial distribution area of the lower ecological spatial carrying capacity was the largest, 1851.03km^2^, accounting for 47.9% of the total land area of Duolun County, mainly distributed in the southwest. The second is the space area with higher coupling capacity, which is 979.95km^2^, accounting for 25.3% of the total land area of Duolun County, and is distributed in the northeast of the county; In 2005, the average ecological spatial carrying capacity of Duolun County was 0.411. The spatial distribution areas of low carrying capacity, low carrying capacity and high carrying capacity in each carrying capacity area were 1077.84–1106.96 km^2^, accounting for 87.3% of the total land area of Duolun County, and widely distributed in the whole county; In 2010, the average ecological spatial carrying capacity of Duolun County was 0.397. The spatial distribution area of lower and higher ecological spatial carrying capacity in each coupling capacity area was large, with a total area of 2945.39km^2^, accounting for 76% of the total land area of Duolun County. The spatial distribution area of high coupling capacity is the lowest, which is 440.52km^2^, accounting for 11.4% of the total land area of Duolun County; The average ecological spatial carrying capacity of Duolun County in 2015 was 0.497, and the spatial distribution area of the lower ecological spatial carrying capacity was the largest, 1765.51km^2^, accounting for 45.7% of the total land area of Duolun County. The second is the spatial distribution area of higher coupling capacity, which is 1162.64km^2^, accounting for 30% of the total land area of Duolun County; The average ecological spatial carrying capacity of Duolun County in 2020 is 0.597. The spatial distribution area of higher and lower ecological spatial carrying capacity in each coupling capacity area is large, with a total area of 2806.86km^2^, accounting for 72% of the total land area of Duolun County. The spatial distribution area of high coupling capacity is the lowest, 195.1km^2^, accounting for 5% of the total land area of Duolun County.

From 2000 to 2020, the ecological spatial carrying capacity of Duolun County increased from 0.441 in 2000 to 0.597 in 2020, showing a downward trend first and then an upward trend on the whole.

It can be seen from Fig 6 that the average comprehensive coupling capacity of PLES in Duolun County in 2000 was 0.482, and the spatial distribution area of higher coupling capacity in each coupling capacity area of PLES was the largest, 1362.68km^2^, accounting for 35% of the total land area of Duolun County, followed by the area of high coupling capacity, 1100.75km^2^, accounting for 28.5% of the total land area of Duolun County; The average comprehensive coupling capacity of PLES in Duolun County in 2005 was 0.365, and the spatial distribution area of the lower coupling capacity in each coupling capacity area of the comprehensive coupling capacity was the largest, which was 2231.293km^2^, accounting for 57.7% of the total land area of Duolun County, mainly distributed in the southern and Northern regions; In 2010, the average comprehensive coupling capacity of PLES in Duolun County was 0.378, and the spatial distribution area of higher coupling capacity in each coupling capacity area of comprehensive coupling capacity was the largest, 1406.32km^2^, accounting for 36.4% of the total land area of Duolun County. The second is the spatial distribution area of high coupling capacity, which is 1096.24km^2^, accounting for 28.4% of the total land area of Duolun County; The average comprehensive coupling capacity of PLES in Duolun County in 2015 was 0.380, and the spatial distribution area of high coupling capacity in each coupling capacity area of PLES comprehensive coupling capacity was the largest, 1822.63km^2^, accounting for 47.2% of the total land area of Duolun County. The second is the spatial distribution area of higher coupling capacity, which is 989.473km^2^, accounting for 25.6% of the total land area of Duolun County; In 2020, the average comprehensive coupling capacity of PLES in Duolun County was 0.215, and the spatial distribution area of the lower coupling capacity of the comprehensive coupling capacity of PLES was the largest, 1571.9.03km^2^, accounting for 40.7% of the total land area of Duolun County. The second is the spatial distribution area of higher coupling capacity, which is 1257.03km^2^, accounting for 32.5% of the total land area of Duolun County.

From 2000 to 2020, the comprehensive carrying capacity of PLES in Duolun County decreased from 0.482 in 2000 to 0.215 in 2020, showing a rapid downward trend.

### 3.3 Spatiotemporal differentiation characteristics of the coupling coordination of PLES

**Fig 8:**
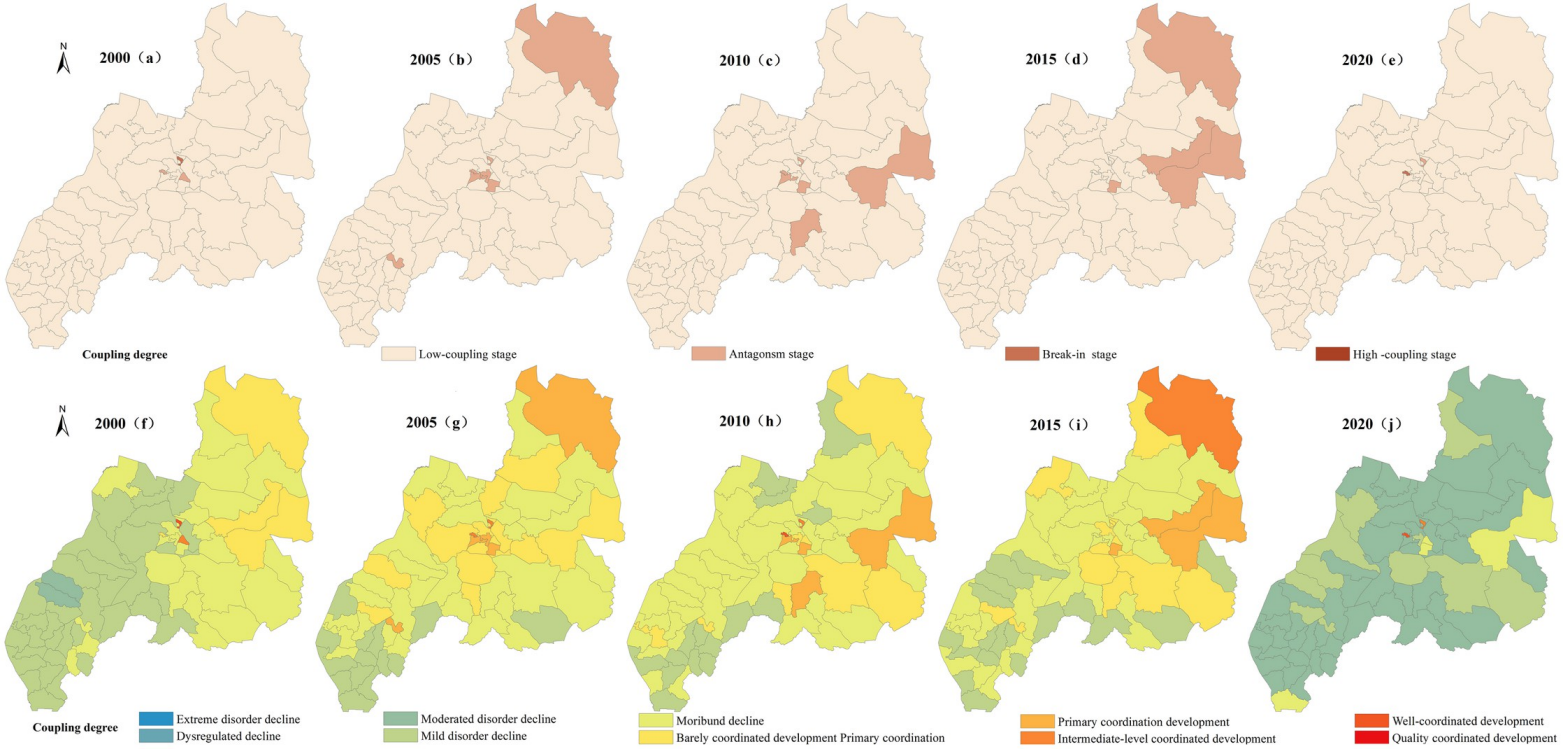
Spatial variation diagram of coupling degree and coupling coordination degree of carrying capacity between production space and living space.

**Fig 9:**
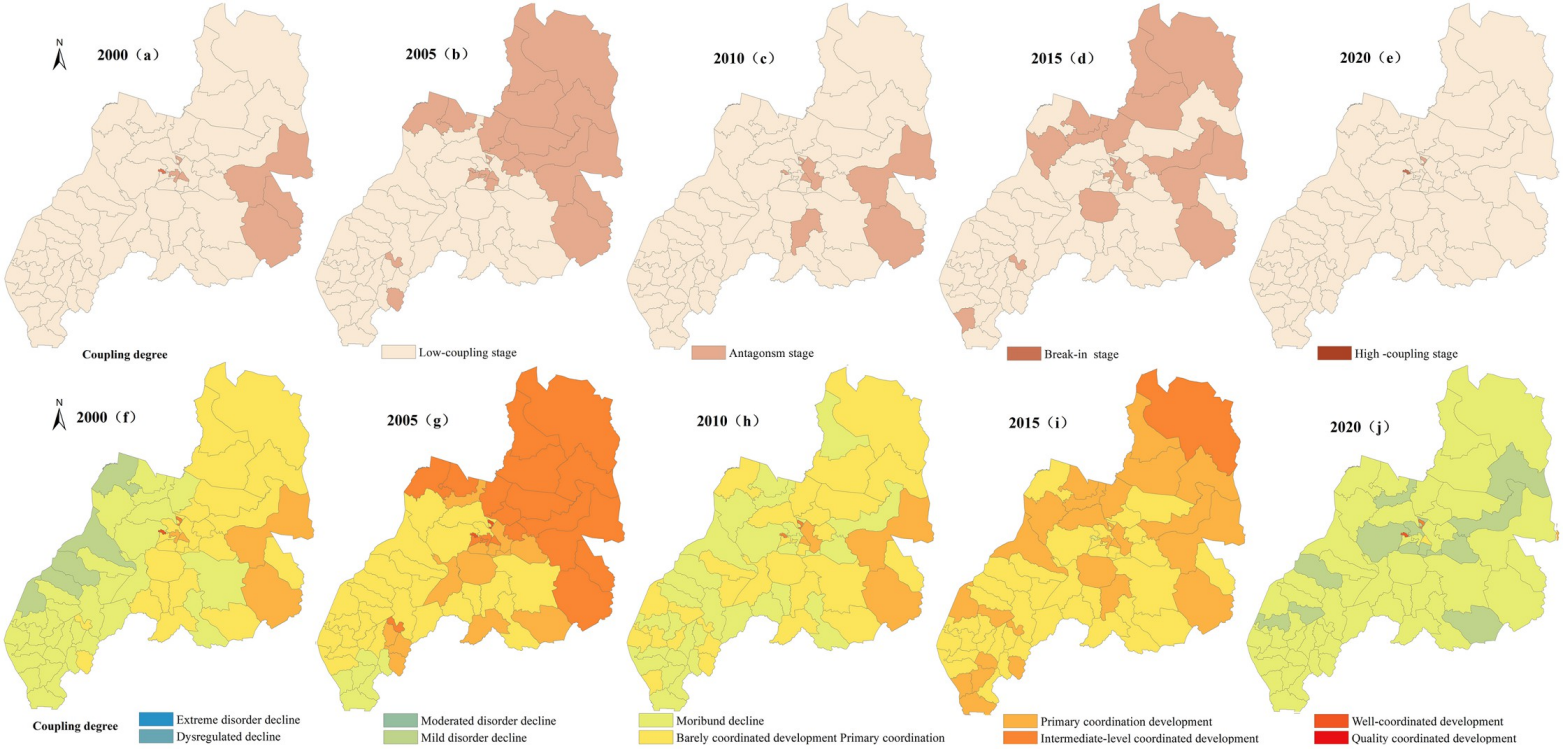
Spatial variation diagram of coupling degree and coupling coordination degree of carrying capacity between ecological and production space.

**Fig 10:**
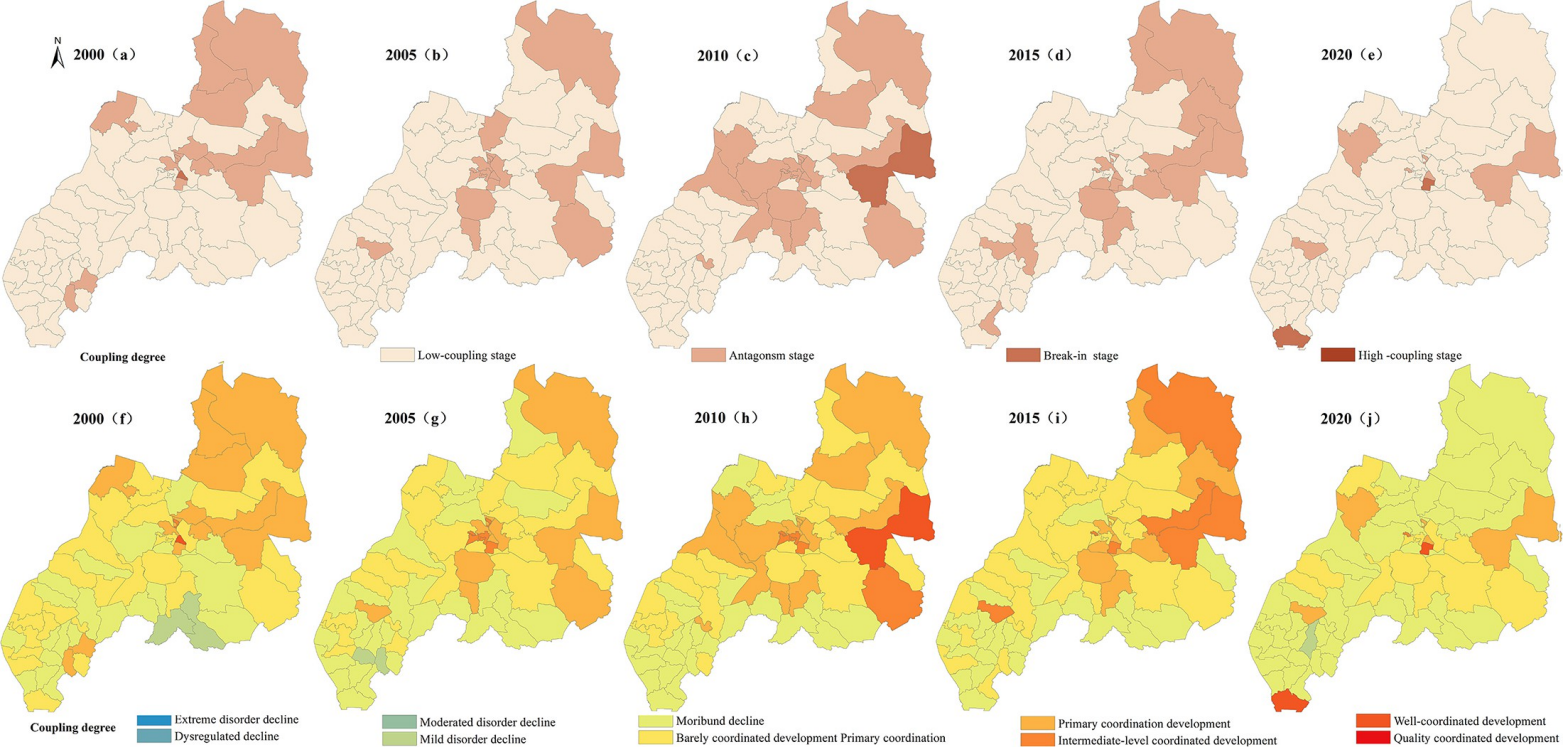
Spatial variation diagram of coupling degree and coupling coordination degree of carrying capacity between ecological and living space.

**Fig 11:**
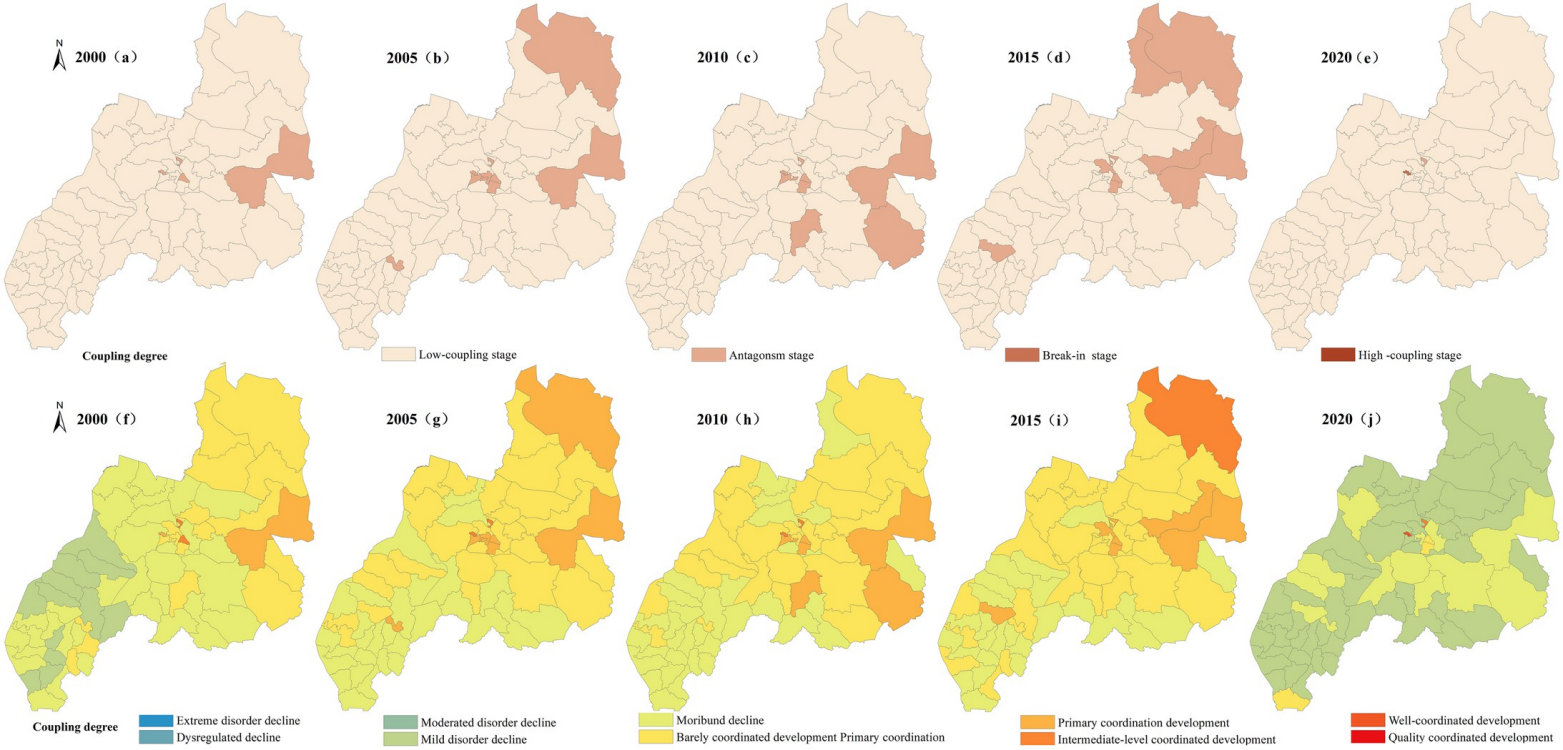
Spatial differentiation of coupling degree and coupling coordination degree of carrying capacity in PLES.

In accordance with existing results [40], the coupling degree C is classified as Low-coupling stage C∈(0,0.35], Antagonism stage C∈(0.35,0.65], Break-in stage C∈(0.65,0.85], High coupling stage C∈(0.85,1], The coupling coordination degree D is divided into Extreme disorder decline D∈(0,0.10], dysregulated decline D∈(0.10,0.20], Moderated disorder decline D∈(0.20,0.30], Mild disorder decline D∈(0.30,0.40], Moribund decline D∈(0.40,0.50], Barely coordinated development Primary coordination D∈(0.50,0.60], Primary coordination development D∈(0.60,0.70], Intermediate-level coordinated development D∈(0.70,0.80], Well-coordinated development D∈(0.80,0.90], Quality coordinated development D∈(0.90,1.0]. Using the manual break point classification method in ArcGIS 10.8 software, the distribution maps of coupling degree and coupling coordination degree of various spaces are produced respectively Figs 8–11.

#### 3.3.1 Characteristics of coupling coordination pattern between production space carrying capacity and living space carrying capacity

**Fig 12:**
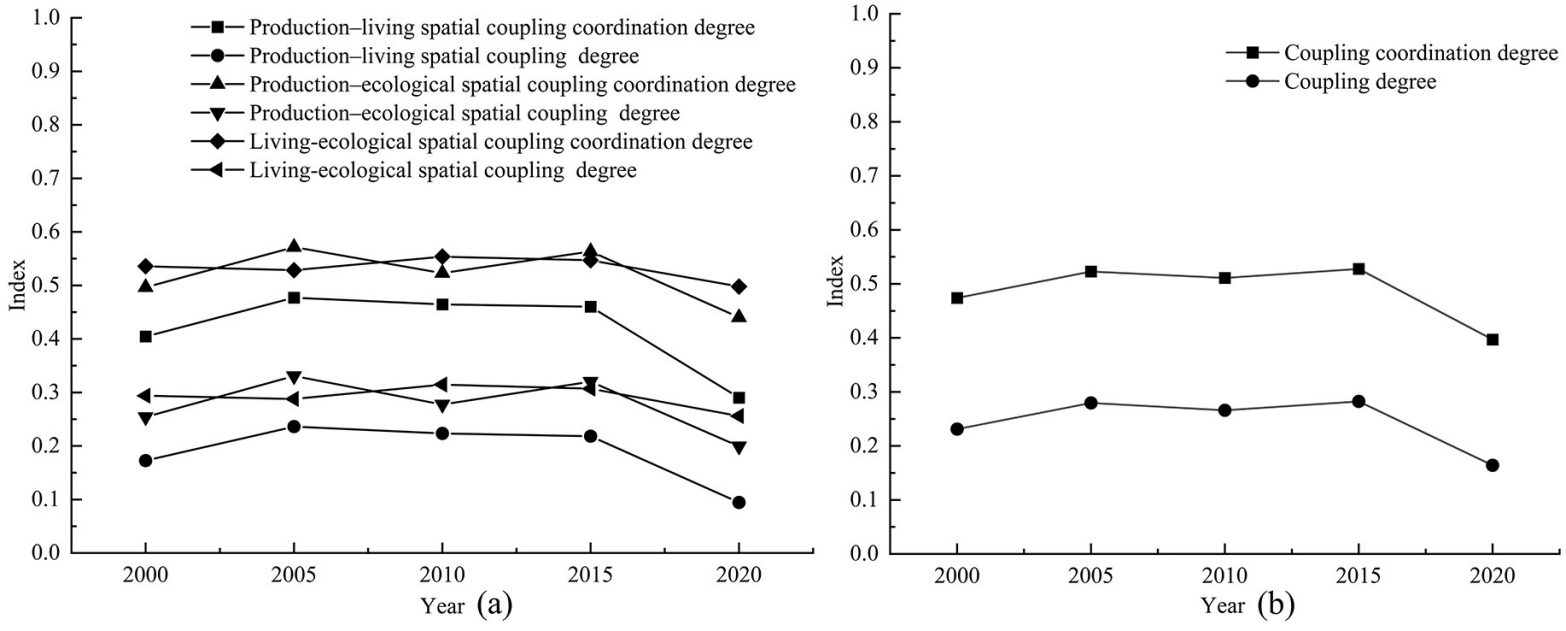
The coupling degree and coupling capacity scheduling change curve of the PLES in Duolun County.

According to the change of coupling degree in Fig 8, in 2000, 2015 and 2020, the areas with low coupling degree of production carrying capacity and living space carrying capacity in Duolun County accounted for 99.8% and 99.9% of the total land area, and the production carrying capacity and living space carrying capacity in Duolun County were basically in a low coupling state. From 2005 to 2010, the coupling degree of production carrying capacity and living space carrying capacity in Duolun County was between 88% and 94% in the low coupling period, and between 6% and 12% in the antagonistic stage. It can be seen from Fig 12(a) that the coupling degree of production carrying capacity and living space carrying capacity in Duolun County from 2000 to 2020 showed a downward trend, and the coupling degree of production carrying capacity and living space carrying capacity in the Northeast was higher than that in the southwest, and was developing towards a low coupling period.

It can be seen from the change of coupling coordination in Fig 7 that the coupling coordination degree of production capacity and living space capacity in Duolun County from 2000 to 2015 belongs to the types of moderated disorder decline, mild disorder decline, moribund decline, barely coordinated development and primary coordination development, but by 2020, it will change to the types of moderated disorder decline, mild disorder decline and moribund decline, that is, the coupling coordination degree of production capacity and living space capacity is decreasing, and the spatial distribution of the coupling coordination degree of production capacity and living space capacity is higher in the Northeast than in the southwest. As shown in Fig 12(a), the level of coordination between production capacity and living space capacity is decreasing.

#### 3.3.2 Characteristics of coupling coordination pattern between production space carrying capacity and ecological space carrying capacity

According to the change of coupling degree in Fig 9, the areas with low coupling degree of production carrying capacity and ecological space carrying capacity in Duolun County accounted for 82% and 94% of the total land area from 2000 to 2015, and the proportion in 2020 was 99%, that is, the production carrying capacity and ecological space carrying capacity in Duolun County were basically in a low coupling state. According to Fig 12(a), the coupling degree of production carrying capacity and ecological living space carrying capacity of Duolun County from 2000 to 2020 showed a downward trend. From 2000 to 2015, the coupling degree of production carrying capacity and ecological spatial carrying capacity was high in the northeast and low in the southwest, and developed to a low coupling period from 2000 to 2020.

In 2000, the coupling coordination degree of production carrying capacity and ecological spatial carrying capacity belonged to the types of mild disorder decline, moribund decline, barely coordinated development and primary coordination development, but by 2020, the coupling coordination degree of production carrying capacity and ecological spatial carrying capacity evolved into the types of dysregulated decline, moderated disorder decline, mild disorder decline and moribund decline. In terms of spatial distribution, the coupling coordination degree of production carrying capacity and ecological spatial carrying capacity is higher in the Northeast than in the southwest, and its coupling coordination level is also decreasing year by year. In 2000, the coupling coordination level of production carrying capacity and ecological spatial carrying capacity belonged to the level of barely coordinated development, primary coordination development and imminent maladjustment, but by 2020, it had evolved into a coupling coordination type dominated by moribund decline, and the level of mutual coordination between the two was also declining.

According to the change of coupling degree in Fig 10, the coupling degree of Duolun County’s life carrying capacity and ecological space carrying capacity from 2000 to 2020 belongs to the stage of low-coupling stage and antagonism stage, and its total area accounts for 99% to 100% of the total land area. The coupling degree of living space carrying capacity and ecological space carrying capacity is at a relatively low level of development, and the correlation between the two is not strong. The strength of the interaction between living space carrying capacity and ecological space carrying capacity is gradually weakening. It can be seen from Fig 12(a) that the coupling degree of production carrying capacity and ecological space carrying capacity in Duolun County from 2000 to 2020 has the characteristics of high in the northeast and low in the southwest, and the coupling degree of production carrying capacity and ecological living space carrying capacity shows a downward trend.

In 2000, the coupling coordination degree of production carrying capacity and ecological spatial carrying capacity belonged to moderated disorder decline, mild disorder decline, moribund decline and barely coordinated development, but by 2020, the coupling coordination degree of production carrying capacity and ecological spatial carrying capacity evolved into moderated disorder decline, mild disorder decline and barely coordinated development. In terms of spatial distribution, the coupling coordination degree of production carrying capacity and ecological spatial carrying capacity is higher in the Northeast than in the southwest. The coupling coordination level is also decreasing year by year, and the mutual coordination level of the two is also decreasing.

#### 3.3.3 Characteristics of coupling coordination pattern of comprehensive carrying capacity of PLES

It can be seen from the change of coupling degree in Fig 11 that the coupling degree of PLES carrying capacity in Duolun County from 2000 to 2015 belongs to the low coupling stage of large area and the antagonistic stage of relatively small area. The coupling degree of the PLES coupling capacity of Duolun County in 2020 belongs to the low coupling stage. The coupling degree of PLES coupling capacity is at a relatively low level of development, and the correlation between the two is not strong, and the strength of PLES coupling capacity is gradually weakening. It can be seen from Fig 12(a) that the coupling degree of PLES coupling capacity in Duolun County from 2000 to 2020 is characterized by high in the northeast and low in the southwest.

In 2000, the coupling coordination degree of PLES carrying capacity all belonged to the stage of mild disorder decline, moribund decline, barely coordinated development, primary coordination development and well-coordinated development, but by 2020, the coupling coordination degree of PLES carrying capacity has evolved into the type of mild disorder decline, moribund decline and barely coordinated development. In terms of spatial distribution, the coupling coordination degree of PLES carrying capacity is higher in the Northeast than in the southwest, and its coupling coordination level is also decreasing year by year, and the mutual coordination level of the two is also decreasing.

It can be seen from Fig 12(b) that the spatial carrying capacity of the PLES in Duolun County from 2000 to 2020 showed a very low coupling effect, and the coupling degree coordination degree of all village units was less than 0.5. The coupling degree of all administrative villages did not show running in and coordination state, with a small change range and a downward trend, indicating that the mutual promotion between PLES carrying capacity tended to weaken. This shows that the coupling coordination degree of PLES in Duolun County is unstable, so it is necessary to make rational use of land space and take corresponding land space optimization measures to improve the coupling coordination of PLES. From 2000 to 2020, the coupling degree of PLES function in Duolun County was much greater than that of coupling coordination, indicating that the degree of interaction among production space carrying capacity, living space carrying capacity and ecological space carrying capacity was much higher than that of coordination among production space carrying capacity, living space carrying capacity and ecological space carrying capacity.

### 3.4 Diagnosis of factors that hinder the carrying capacity of the PLES

#### 3.4.1 Obstacle degree of criterion layer

**Fig 13:**
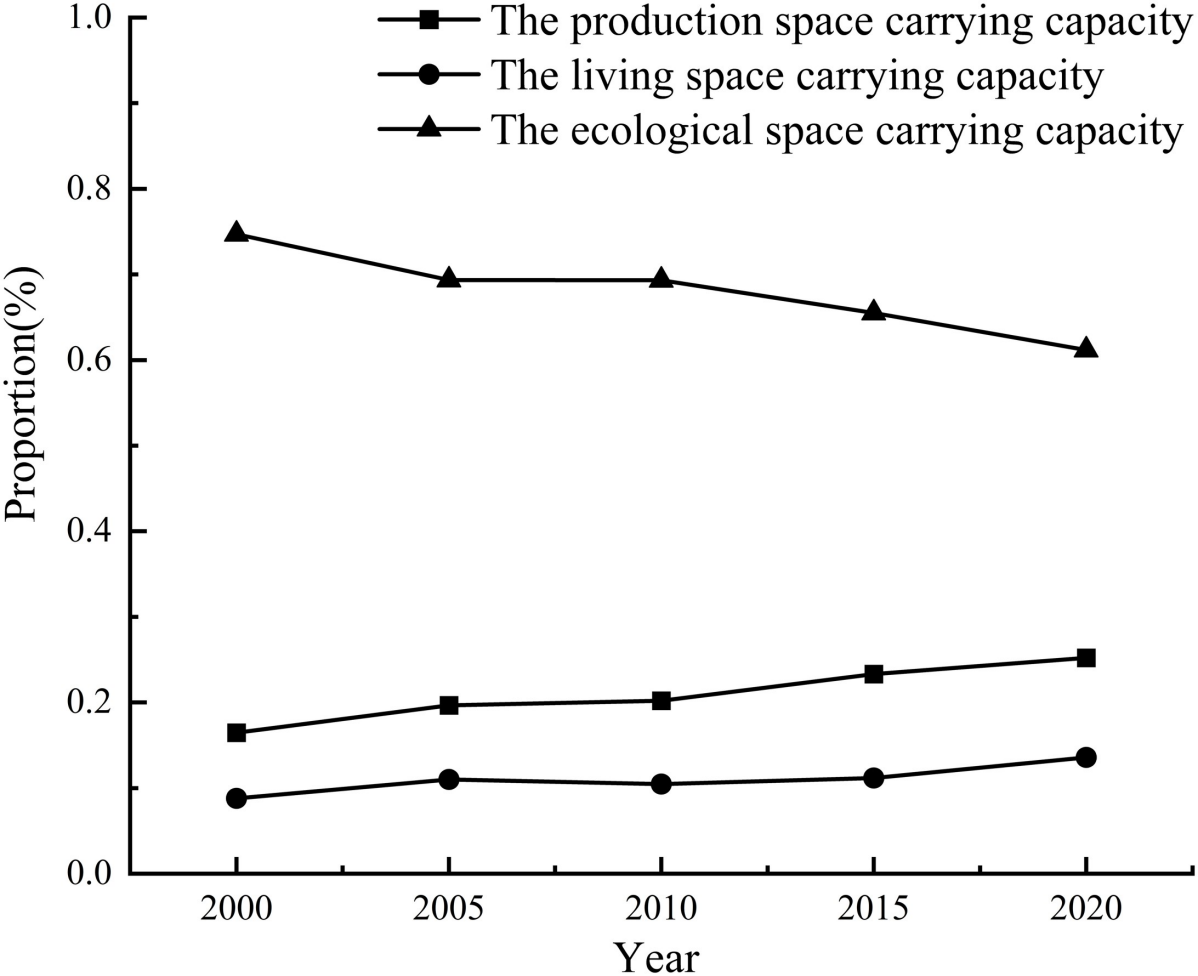
Obstacle degree of the PLES carrying capacity criterion layer in Duolun County.

It can be seen from Fig 13 that there are significant differences in the obstacle degree and its change of the PLES spatial carrying capacity criterion layer in Duolun County, Inner Mongolia from 2000 to 2020. The obstacle degree of production space carrying capacity and living space carrying capacity increased by 0.09 and 0.048 respectively, indicating that the obstacle degree of production space carrying capacity and living space carrying capacity of the selected influencing factors increased. The obstacle degree of ecological space carrying capacity decreased by 0.14, indicating that the obstruction effect of the selected influencing factors on the production space carrying capacity was significantly weakened, which indicates that there is a certain contradiction between the development and utilization of land space and the protection of ecological space. From the obstacle degree value, it can be seen that the obstacle degree of the ecological space carrying capacity is much higher than that of the production space carrying capacity and the living space carrying capacity, which indicates that the ecological space carrying capacity has become the main impact index of the sustainable impact of the three living space carrying capacity. It can be seen that the development of agriculture and animal husbandry and the construction of road network in Duolun County of Inner Mongolia in the past 20 years have increased the pressure of ecological carrying capacity and hindered the improvement of ecological carrying capacity.

#### 3.4.2 Obstacle degree of index layer

**Fig 14:**
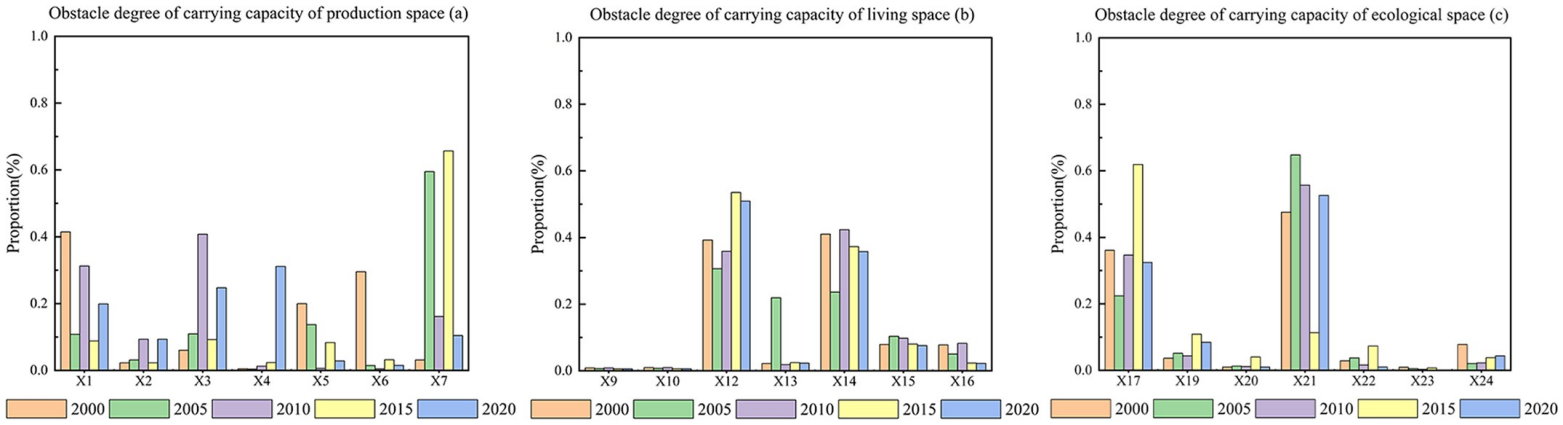
Obstacle degree of the PLES carrying capacity in Duolun County.

**Fig 15:**
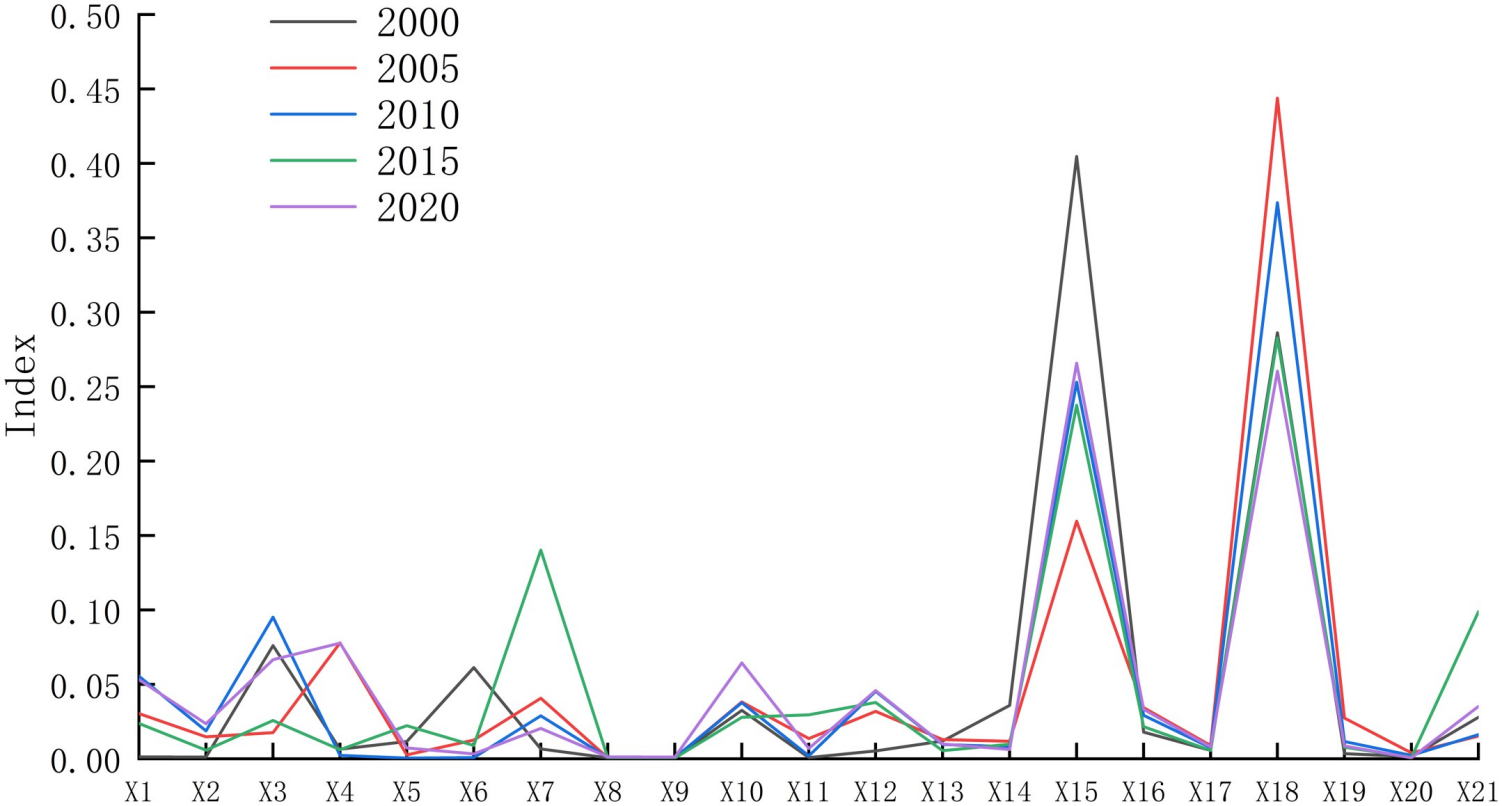
The average obstacle degree of the PLES carrying capacity indicators in Duolun County.

It can be seen from Fig 14(a) that carrying capacity has the greatest obstacle to the carrying capacity of production space, followed by GDP that is, carrying capacity GDP is the main obstacle restricting the carrying capacity of production space. From 2000 to 2020, the obstacle degree of GDP to the carrying capacity of production space increased, while the obstacle degree of carrying capacity decreased rapidly. It can be seen from Fig 14(b) that the scale of construction land has the greatest obstacle to the carrying capacity of living space, followed by the total population. The scale of construction land, total population and per capita disposable are the main obstacles restricting the carrying capacity of living space. From 2000 to 2020, the barriers of construction land scale, total population and per capita disposable to the carrying capacity of living space showed a rapid increasing trend. It can be seen from Fig 14(c) that the degree of desertification has the greatest obstacle to the ecological spatial carrying capacity, followed by the degree of precipitation, that is, the degree of desertification and precipitation are the main obstacles restricting the ecological spatial carrying capacity. It can be seen from Fig 15 that from 2000 to 2020, the obstacle degree of desertification degree to the carrying capacity of living space decreased, while the obstacle degree of precipitation increased. The obstacle degree of soil organic matter to the comprehensive carrying capacity of the PLES is the largest, followed by the obstacle degree of precipitation, that is, soil organic matter and precipitation are the main obstacle factors restricting the comprehensive carrying capacity of the PLES. From 2000 to 2020, the obstacle degree of soil organic matter to the comprehensive carrying capacity of the the PLES increased, while the obstacle degree of precipitation showed a weak decreasing trend.

## 4 Discussion

### 4.1 Dynamic evolution pattern of the PLES

From 2000 to 2020, the area of single function space dominated by ecological function and production function in Duolun County of Inner Mongolia showed a downward trend, which may be related to the reduction of the area of dry land [41] and irrigated land [42]. Since 2000, the implementation of "Beijing Tianjin sandstorm source area management", "returning farmland to forest and Grassland", "eliminating wasteland", "ecological migration", ecological protection and restoration of Inner Mongolia Plateau and other projects [43] has greatly increased the forest land area, rapidly reduced the cultivated land area [44], and improved the ecological environment [45]. The living space area shows a slight upward trend, which is related to the increase in the construction land area of the ecological immigrant village in the Duolun Naoer area of the county center [35]. The distribution area of the PLES, mainly based on the combination of production and ecological functions, is showing a rapid growth trend, which may be related to the growth of other forest and artificial grassland areas.

### 4.2 The dynamic evolution pattern of the carrying capacity of the PLES

The production space carrying capacity of administrative villages in Duolun County showed a weak decreasing trend. The Hunshandake Sandy Land in the north of Duolun County has a bad ecological environment and serious land desertification. The ecological immigration policy has been implemented since 2000. The ecological immigration policy has reduced the local population density, reduced the labor force, and reduced the production space carrying capacity. The implementation of the ecological resettlement project has transformed a large area of rural construction land into woodland and grassland, reducing the area of living space [46]. Reduced its carrying capacity. In addition, rural roads have no living space function, and the living function of rural homestead is very high. The reduction of rural road construction and rural homestead area will directly lead to the reduction of living space carrying capacity. The comprehensive carrying capacity of ecological space showed a weak growth trend, which may be related to the reduction of land desertification area and precipitation. The comprehensive carrying capacity of the PLES decreased significantly, which may be related to the decrease of soil organic matter content. The area with high carrying capacity of production space and living space in Duolun County of Inner Mongolia has a larger grassland area, while the area with low carrying capacity of production space and living space has a larger proportion of forest land area. The increase in the number of livestock has promoted the increase in the output value of the primary industry [47]. After returning farmland to forest and grassland, the area of grassland and woodland increased, the production function of woodland and grassland was lower than that of cultivated land, and the production function of cultivated land was the highest. The reduction of cultivated land area reduced the production function of cultivated land. Duolun County is located in the ecotone of agriculture and animal husbandry in the north, which is affected by the vulnerability of the ecosystem in the arid and semi-arid area [48], reducing the comprehensive carrying capacity of its the PLES. Because the content of soil organic matter in the northeast of Duolun County is higher than that in the southwest, it leads to the pattern characteristics of "high in the northeast and low in the southwest" of the comprehensive carrying capacity of the PLES.This is related to the ecological protection and construction projects vigorously implemented by the local government.

### 4.3 The coupling and coordinated evolution pattern of the PLES

Duolun County is located on the southern edge of the Hunshandak Sandy Land and is a typical agricultural pastoral economic zone in the northern agricultural pastoral transitional zone. Due to the relatively fragile ecological environment in Duolun County, the development model that sacrifices the environment in the process of socio—economic development has made it difficult for the local ecological environment to bear the enormous pressure brought about by rapid economic development. Before 2000, animal husbandry was the main focus, but due to overloading and overgrazing, the area of desertified land increased year by year, farmland and grassland degraded severely, and crop yields decreased year by year. Faced with a harsh ecological environment, since 2000, Duolun County has increased its efforts to control desertified and degraded land, implementing a policy of banning grazing and planting trees for all livestock species, resulting in a significant increase in the area of other forest and shrub forests, as well as other grasslands in various land use types. The production function of other forests, shrublands, and grasslands is weak, while the ecological function is strong.The strategy of returning farmland back to forests has resulted in a decline. In addition, the organic matter content in the local soil is relatively low, and there is a slight decrease in precipitation. These human and natural factors ultimately lead to production carrying capacity, living carrying capacity, ecological carrying capacity, and low comprehensive carrying capacity of the PLES organisms. The low carrying capacity level [49] limits the improvement of the coupling coordination of the PLES spatial carrying capacity in Duolun County.

### 4.4 Obstacles to the evolution of the PLES

Duolun County is located in the northwest monsoon grassland climate zone, with cold and dry climate, barren land, inconvenient transportation, backward market, low level of animal husbandry, small output of agricultural products and lack of support from other industries, which has brought obstacles to the sustainable development of local agriculture and animal husbandry. The higher the livestock carrying capacity, the lower the vegetation coverage. From 2000 to 2020, with the decline of livestock carrying capacity in Duolun County, the degree of obstruction to the carrying capacity of production space and living space decreased year by year, while the degree of obstruction to the carrying capacity of ecological space increased year by year [50]. The larger the scale of construction land, the higher the density of road network. The density of road network plays an important role in the development of agriculture and animal husbandry, and has a great impact on the transportation and operation costs of living space. With the increase of precipitation, the vegetation coverage is gradually increasing [51]. Affected by climate change and human factors, the desertified land area of Duolun County in 2000 was 728.32km^2^, accounting for 18.69% of the land area [52]. In 2020, the land desertification area will be reduced to 144.95km^2^, accounting for 3.8% of the land area. The reduction of the degree of desertification directly leads to the weakening of the obstacle degree of the degree of desertification to the ecological space carrying capacity. The obstacle degree of soil organic matter to the comprehensive carrying capacity of the PLES is the largest, followed by the obstacle degree of precipitation, that is, soil organic matter and precipitation are the main obstacle factors restricting the comprehensive carrying capacity of the PLES.That is, natural factors have a significant impact on the carrying capacity of PLES space. In the future, we should continue to protect the ecological environment and ensure the ecological security of Beijing Tianjin Hebei region.

This study uses multi-source data to explore the evolution characteristics and influencing factors of the carrying capacity of rural PLES from the perspective of PLES, which has important guiding value for the sustainable use of land space at the rural level. However, to a certain extent, it is limited by the availability of data, which may lead to the deviation of the results of PLES bearing capacity data. It is hoped that the existing research results can be improved and improved through higher precision data in future research work.

## 5 Conclusions

1. From 2000 to 2020, the area of single function area in the the PLES functions of administrative villages in Duolun County, Inner Mongolia, accounted for a large proportion, and the single function area was mainly ecological space area. The distribution area of ecological space and production space showed a decreasing trend, while the area of living space showed a weak growth trend. The land spatial distribution area based on the combination of production and ecological functions showed a rapid growth trend.
2. From 2000 to 2020, the production space carrying capacity, living space carrying capacity, and the comprehensive living space of the administrative villages in Duolun County in Inner Mongolia showed a weak decreasing trend, while the ecological space carrying capacity showed a significant upward trend.
3. From 2000 to 2020, the coupling degree and coupling coordination degree of the production space and living space carrying capacity, production space and ecological space carrying capacity, living space and ecological space carrying capacity, and the comprehensive carrying capacity of the PLES in the administrative villages of Duolun County in Inner Mongolia showed a slow downward trend year by year, showing the spatial distribution characteristics of "high in the northeast and low in the southwest".
4. From 2000 to 2020, the obstacle degree of production space carrying capacity and living space carrying capacity of administrative villages in Duolun County, Inner Mongolia increased, while the ecological space decreased. Livestock carrying capacity is the most important obstacle restricting the carrying capacity of production space. The scale of construction land has the greatest obstacle to the carrying capacity of living space, the degree of desertification has the greatest obstacle to the carrying capacity of ecological space, and soil organic matter has the greatest obstacle to the comprehensive carrying capacity of the PLES.

Using multi-source data, this paper reveals the spatio-temporal heterogeneity and coupling coordination of carrying capacity of PLES, which provides a scientific basis for the formulation of regional land spatial optimization. Subsequently, we can combine high-resolution data to explore the undisclosed spatial heterogeneity information, and provide more accurate information and scientific methods for management decision-making.
